# Dynamic changes of MMP-9 plasma levels correlate with JCV reactivation and immune activation in natalizumab-treated multiple sclerosis patients

**DOI:** 10.1038/s41598-018-36535-5

**Published:** 2019-01-22

**Authors:** Marco Iannetta, Maria Antonella Zingaropoli, Tiziana Latronico, Ilaria Pati, Simona Pontecorvo, Carla Prezioso, Valeria Pietropaolo, Antonio Cortese, Marco Frontoni, Claudia D’Agostino, Ada Francia, Vincenzo Vullo, Claudio Maria Mastroianni, Grazia Maria Liuzzi, Maria Rosa Ciardi

**Affiliations:** 1 Department of Public Health and Infectious Diseases, Sapienza University, Rome, Italy; 2 Department of Biosciences, Biotechnology and Biopharmaceutics, Aldo Moro University, Bari, Italy; 3 Department of Human Neuroscience, Multiple Sclerosis Center, Sapienza University, Rome, Italy

## Abstract

The aim of the study was to investigate the changes of matrix metalloproteinase (MMP)-2 and MMP-9 plasma levels during natalizumab treatment and their correlation with JC virus (JCV) reactivation and T-lymphocyte phenotypic modifications in peripheral blood samples from 34 relapsing-remitting multiple sclerosis (RRMS) patients. MMP-9 levels were assessed by zymography in plasma samples. JCV-DNA was detected through quantitative real time PCR in plasma samples. T-lymphocyte phenotype was assessed with flow cytometry. MMP-9 plasma levels resulted increased from 12 to 24 natalizumab infusions. Stratifying plasma samples according to JCV-DNA detection, MMP-9 plasma levels were significantly increased in JCV-DNA positive than JCV-DNA negative samples. MMP-9 plasma levels resulted positively correlated with JCV viral load. CD4 immune senescence, CD8 immune activation and CD8 effector percentages were positively correlated to MMP-9 plasma levels, whereas a negative correlation between CD8 naïve percentages and MMP-9 plasma levels was found. Our data indicate an increase of MMP-9 plasma levels between 12 and 24 natalizumab infusions and a correlation with JCV-DNA detection in plasma, T-lymphocyte immune activation and senescence. These findings could contribute to understand PML pathogenesis under natalizumab treatment, suggesting a potential role of MMP-9 as a predictive marker of PML in RRMS patients.

## Introduction

Multiple sclerosis (MS) is a chronic inflammatory disease of the central nervous system (CNS) characterized by demyelination and axonal loss^1^ and represents a prominent cause of neurological disability among young adults^2^. Several studies suggest that brain inflammation during MS is initiated and sustained by T-lymphocyte migration across the blood-brain barrier (BBB) and is considered to be mediated by T helper (Th)1 cells, while Th2 cells seem to have protective and repair-promoting functions^3^. Accordingly, increased levels of Th1 cells are found in the brain, blood and CSF of MS patients^3^.

Based on current studies, CD4^+^CD28^−^ T-cells play a critical role in the pathogenesis of autoimmune diseases, such as MS^4^. CD28 is constitutively expressed on the surface of more than 95% of CD4^+^ T-lymphocytes and regulates Th1/Th2 differentiation and proliferation, including cell-cycle progression and susceptibility to apoptotic cell death^5^. The loss of CD28 expression on both CD4+ and CD8^+^ T-cells was identified as a reliable biological marker of aging of the immune system^6^. CD4^+^CD28^−^ T-lymphocytes percentages are increased in MS patients^4^. The presence of these cells early in the disease, suggests that CD4^+^CD28^−^ T-lymphocytes may be actively involved in MS pathogenesis^6^. Besides thymic involution, several other mechanisms could contribute to the age-inappropriate high frequencies of CD4^+^CD28^−^ T-cells in MS patients, such as viral infections, which can cause massive proliferation and aging of peripheral T cells^7^. Moreover HLA-DR and CD38 are considered phenotypic markers of chronic T-lymphocyte immune activation, and were used in the settings of neuroinflammatory diseases to assess CD4 and CD8 activation status^8^.

During MS, the immune cell migration into the CNS depends on BBB permeability, which can be increased by dysregulation of matrix metalloproteinase (MMP) activity. In particular, MMP-2 (gelatinase A, 72 kDa type IV collagenase) and MMP-9 (gelatinase B, 92 kDa type IV collagenase) have been extensively studied in MS^9^. Experimental results demonstrated the intrathecal synthesis of MMP-9 in MS^10^ and the increase of its levels in cerebrospinal fluid (CSF) and serum of MS patients affected by the relapsing-remitting (RR) form compared to the primary progressive form and healthy controls^11,12^. The elevated CSF and serum concentrations of MMP-9 were associated with magnetic resonance imaging (MRI) and clinical evidence of disease activity^13,14^ and evolution^15^ suggesting that MMP-9 represents a biomarker of disease activity^10^ and a putative therapeutic target for MS^16^. In this respect, it has been demonstrated that IFN-β reduces T-lymphocyte transmigration by interfering with the production of MMP-9^17^ and that the treatment of RRMS patients with IFN-β reduces serum MMP-9 concentrations and ameliorates the course of the disease^18^.

Currently, natalizumab (Tysabri, Biogen Idec Inc., Cambridge, Massachusetts, USA), a humanized anti-α4 integrin monoclonal antibody, is one of the most effective treatment for RRMS^19^. Natalizumab blocks the interaction between α4β1 integrin (very late antigen-4 [VLA-4]), expressed on the surface of T-lymphocytes, and the vascular-cell adhesion molecule 1 (VCAM-1), expressed on the surface of vascular endothelial cells in brain and spinal cord blood vessels, limiting T-lymphocyte migration into the CNS^20^. The reduced T-cells migration across the BBB, is thought to be the reason for the increased risk of developing progressive multifocal leukoencephalopathy (PML), caused by John Cunningham Virus (JCV) reactivation^21^.

Khademi *et al*. demonstrated that CSF MMP-9 mean levels were reduced after 12 months of natalizumab treatment in 7 MS patients^22^. Accordingly, other authors showed that serum protein levels of MMP-9 were reduced in RRMS patients after 12 months of natalizumab treatment^23^. While MMP-9 is predominantly upregulated in inflammatory conditions, MMP-2 is constitutively expressed in the brain^24^.

The aim of the present study was to investigate the changes in plasma MMP-2 and MMP-9 levels during natalizumab treatment and their correlation with JCV detection, T-lymphocyte immune activation and immune senescence markers. Our findings suggest a potential pathogenic role of MMP-9 in the development of PML during natalizumab treatment and its possible use as a marker of JCV reactivation.

## Results

### Detection of MMP plasma levels by zymography

MMP-2 and MMP-9 plasma levels were measured by zymography on gelatin-copolymerized gels in 116 plasma samples from 34 RRMS patients stratified according to natalizumab infusion number.

**Figure 1:**
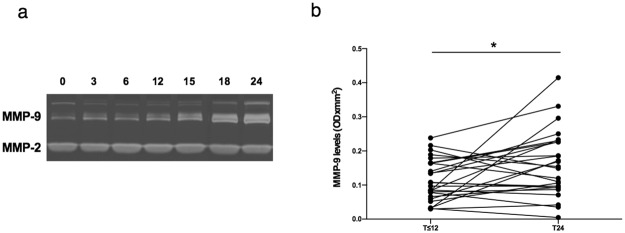
MMP-9 levels in plasma samples from RRMS patients according to natalizumab infusion number. MMP-9 plasma levels were assessed by zymography in 116 plasma samples from 34 RRMS patients under natalizumab treatment. Representative zymographic gel. MMP‐2 and MMP‐9 were identified by their apparent molecular mass of 67 and 92 kDa, respectively. The zymographic gel represents MMP-9 and MMP-2 plasma level analysis of samples from the same patient: starting from the first left lane, plasma samples collected at 0, 3, 6, 12, 15, 18 and 24 natalizumab infusions are represented, respectively. The gel represents the grouping of lanes cropped from different parts of the same gel (**a**). The full-length gel is included in the Supplementary Fig. S1a. Longitudinal analyses of plasma samples collected from 26 RRMS patients within 12 (T ≤ 12) and at 24 (T24) natalizumab infusions, after quantitation of MMP‐2 and MMP‐9 levels through scanning densitometry and computerize analysis of zymographic gels. For T ≤ 12 group, all samples collected at T0 (before first natalizumab infusion) were included in the analysis. When a T0 sample was not available, the first sample collected within 12 natalizumab infusions was considered. IQR: interquartile range. MMP-9 median level [IQR]: 0.10 [0.06–0.16] for T ≤ 12 and 0.15 [0.09–0.23] for T24. *p < 0.05 (Wilcoxon test) (**b**).

A representative gel from an individual patient is shown in Fig. 1a. No variations were observed for plasma levels of MMP-2, which is constitutively expressed in body fluids and was used as an internal control of sample processing. By contrast, MMP-9 levels increased in plasma accordingly with natalizumab infusion number.

The longitudinal analysis, performed in 26 RRMS patients for whom two samples of plasma were available, collected within 12 (T ≤ 12) and at 24 natalizumab infusions (T24), showed a statistically significant increase in MMP-9 plasma levels from T ≤ 12 to T24 (Wilcoxon, p = 0.036) (Fig. 1b).

A bimodal trend in MMP-9 plasma levels was observed from the analysis of the 116 plasma samples collected from all the RRMS patients stratified according to natalizumab infusion number. Specifically, no differences were found in patients after 3, 6 and 12 natalizumab infusions. Conversely, MMP-9 plasma levels increased after 15, 18 and 24 natalizumab infusions (Spearman, ρ = 0.264 and p = 0.024) (Supplementary Fig. S1a). Moreover, the same bimodal trend was observed for MMP-9/MMP-2 ratio (Table 1).

**Table 1:** MMP plasma and CD8 immune activation levels in RRMS patients and healthy donors.

	HD	RRMS						
		Natalizumab infusion number						
		0	3	6	12	15	18	24
MMP-9	0.07 [0.01–0.14]	0.10 [0.06–0.16]	0.08 [0.06–0.13]	0.09 [0.08–0.14]	0.08 [0.05–0.15]	0.11 [0.08–0.16]	0.12 [0.06–0.22]	0.15 [0.09–0.23]
MMP-2	0.06 [0.04–0.07]	0.08 [0.06–0.09]	0.07 [0.06–0.09]	0.07 [0.07–0.09]	0.07 [0.06–0.09]	0.08 [0.07–0.09]	0.082 [0.08–0.10]	0.07 [0.06–0.09]
MMP-9/MMP-2	1.14 [0.73–1.77]	1.61 [0.80–2.28]	1.44 [0.62–2.18]	1.35 [1.07–1.93]	1.32 [0.66–2.16]	1.38 [1.05–2.88]	1.46 [0.62–2.68]	1.90 [1.24–2.92]
CD8 HLA-DR+CD38+	0.95 [0.64–1.9]	1.59 [0.94–2.24]	4.17 [2.60–5.71]	2.93 [1.49–4.34]	1.97 [1.32–3.54]	3.21 [2.20–4.96]	2.25 [0.81–3.84]	2.95 [1.64–5.44]

MMP levels are expressed as OD × mm^2^. CD8 immune activation is expressed as percentage of CD8.

HD: heathy donors. RRMS: relapsing-remitting multiple sclerosis. Data are represented as median values and [interquartile range].

MMP-9 plasma levels were higher in untreated RRMS patients than HD (Supplementary Fig. S1b), even though the difference was not statistically significant (Mann-Whitney, p > 0.05). No differences were found between male and females or considering previous therapies.

### Detection of JCV-DNA

**Figure 2:**
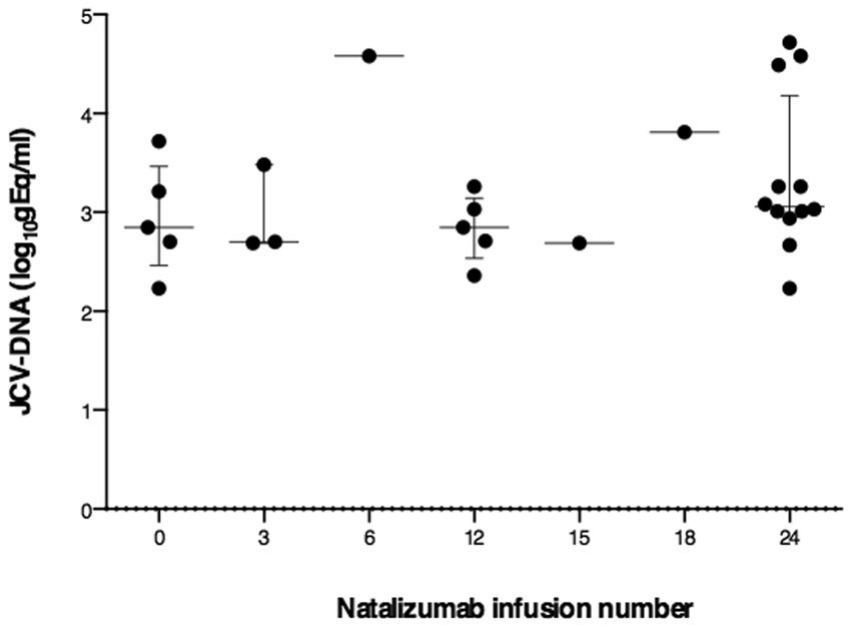
JCV-DNA viral loads according to natalizumab infusion number. JCV viral loads in 28 positive plasma samples during natalizumab treatment are represented. No statistical differences were found (one-way ANOVA test for non-parametrical data, Kruskal-Wallis). Results were expressed as log_10_gEq/ml. Data are shown as median (lines) and interquartile range (whiskers).

Plasma samples from all RRMS patients were analyzed for the detection of JCV-DNA with a real time PCR system. Considering the whole cohort, JCV-DNA was detected in 24.1% (28/116) of plasma samples. Considering the stratification according to natalizumab infusion number, JCV-DNA was detected in 27.8% (5/18), 27.3% (3/11), 7.1% (1/14), 19.2% (5/26), 10% (1/10), 12.5% (1/8) and 41.4% (12/29) of plasma samples at 0, 3, 6, 12, 15, 18 and 24 natalizumab infusions, respectively. As reported in Fig. 2, no statistically significant differences were found in JCV viral loads after grouping patients according to natalizumab infusion number.

### Evaluation of MMP-9 plasma levels according to JCV-DNA detection

**Figure 3:**
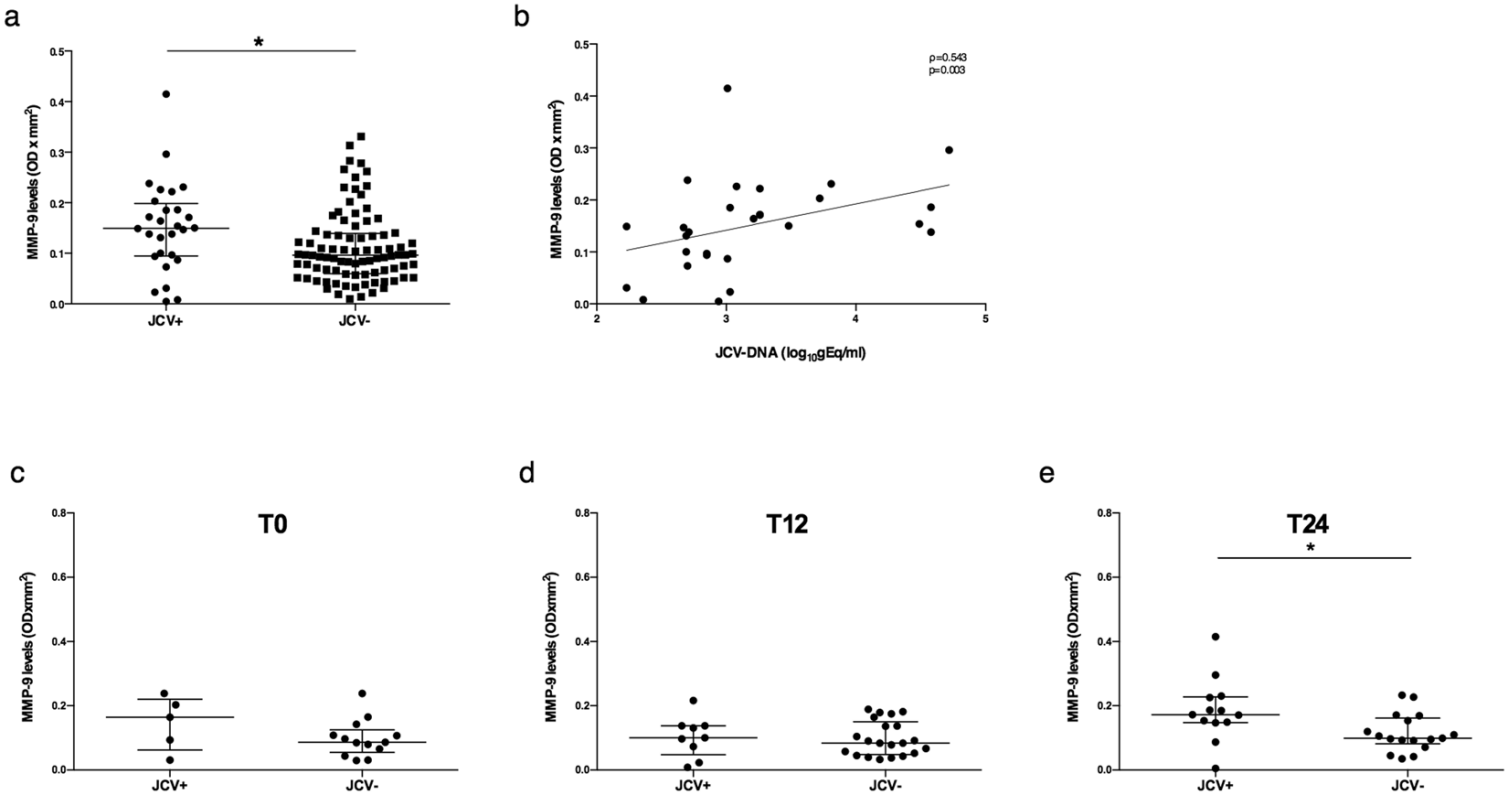
MMP-9 plasma levels in JCV-DNA positive and negative samples and correlation to JCV viral load. MMP-9 plasma levels detected in 116 samples from 34 RRMS patients were higher in the JCV-DNA positive (JCV+) (n = 28) than JCV-DNA negative (JCV−) (n = 88) samples. Data are shown as median (lines) and interquartile ranges (whiskers). OD: optical density (**a**). MMP-9 plasma levels were positively correlated to JCV viral load in JCV+ samples (n = 28). Correlation was performed using Spearman test (Spearman coefficient [ρ] and statistical significance [p] are reported in the graphics). Linear correlation was evaluated by using the regression test, R^2^ = 0.156, p = 0.037 (**b**). Samples (n = 78) were stratified into three groups: T0 (before the first natalizumab infusion) (n = 18); T12 (within the first year of treatment) (n = 30); T24 (during the second year of treatment) (n = 30). No differences were found in MMP-9 plasma levels comparing JCV+ and JCV− samples at T0 (**c**) and T12 (**d**). MMP-9 levels resulted increased in JCV+ compared to JCV− samples for T24 group (**e**). Data are shown as median (lines) and interquartile range (whiskers). JCV+: JCV-DNA positive samples, JCV−: JCV-DNA negative samples. *p < 0.05 (Wilcoxon test).

MMP-9 plasma levels were evaluated after stratifying samples in JCV-DNA positive (JCV+) or negative (JCV−). The explorative analysis performed considering all the 116 samples independently from the natalizumab infusion number, showed increased MMP-9 levels in JCV+ (median values [IQR]: 0.150 [0.095–0.199]) compared to JCV− samples (0.097 [0.060–0.139]) (Mann-Whitney, p = 0.015) (Fig. 3a). Moreover, MMP-9 plasma levels resulted directly correlated with JCV viral load in JCV+ samples (Spearman, ρ = 0.543 and p = 0.003) (Fig. 3b).

In order to avoid sampling biases due to inclusion of multiple samples from the same patient at different timepoints in the same analysis, samples of our cohort were stratified into three groups: T0, before the first natalizumab infusion; T12, within the first year of treatment (2, 2 and 26 samples at 3, 6 and 12 natalizumab infusions, respectively); T24, during the second year of treatment (1 and 29 samples at 18 and 24 natalizumab infusions, respectively). After comparing JCV+ and JCV− samples in each group, no differences were found in MMP-9 plasma levels for T0 (median values [IQR]: 0.164 [0.06–0.221] vs 0.086 [0.056–0.125]), (Mann-Whitney, p = 0.27) and T12 (median values [IQR]: 0.100 [0.048–0.138] vs 0.084 [0.049–0.151]), (Mann-Whitney, p = 0.78), (Fig. 3c,d), while MMP-9 plasma levels resulted increased in JCV+ compared to JCV− samples for T24 group (median values [IQR]: 0.172 [0.148–0.228] vs 0.099 [0.082–0.162]), (Mann-Whitney, p = 0.022) (Fig. 3e).

### Evaluation of T-lymphocyte subsets, immune activation and senescence

T-lymphocyte immune activation was evaluated considering CD38 and HLA-DR co-expression, whereas T-lymphocyte immune senescence was evaluated considering the percentages of CD28^−^CD57^+^ cells by flow cytometry. CD4 and CD8 subsets were characterized by flow cytometry considering surface CD45RO and CD27 expression. Briefly, four CD4 and five CD8 subsets were identified as follows: naïve (N): CD27^+^CD45RO^−^, central memory (CM): CD27^+^CD45RO^+^, effector memory (EM): CD27^−^CD45RO^+^, effector (E): CD27^−^CD45RO^−^ and, only for CD8, intermediate (I): CD27^low^CD45RO^−^.

The evaluation of the different CD4 and CD8 subsets confirmed previous results^25^, showing that CD4 immune activation levels were unchanged during natalizumab treatment, whereas CD8 immune activation were increased in RRMS patients with a longer exposition to natalizumab (Table 1). Moreover, a longitudinal analysis was performed, considering those patients with two whole blood samples available (T ≤ 12 and T24 natalizumab infusions, respectively). An increment in CD8^+^HLA-DR^+^CD38^+^ percentages was found from T ≤ 12 to T24 (Wilcoxon, p = 0.004) (Supplementary Fig. S1c). Under natalizumab treatment, no differences in CD4 and CD8 immune senescence levels were observed. CD4 and CD8 subsets were unchanged comparing the groups at different natalizumab infusion numbers.

### Evaluation of immunophenotyping data according to JCV presence and MMP-9 plasma levels

**Figure 4:**
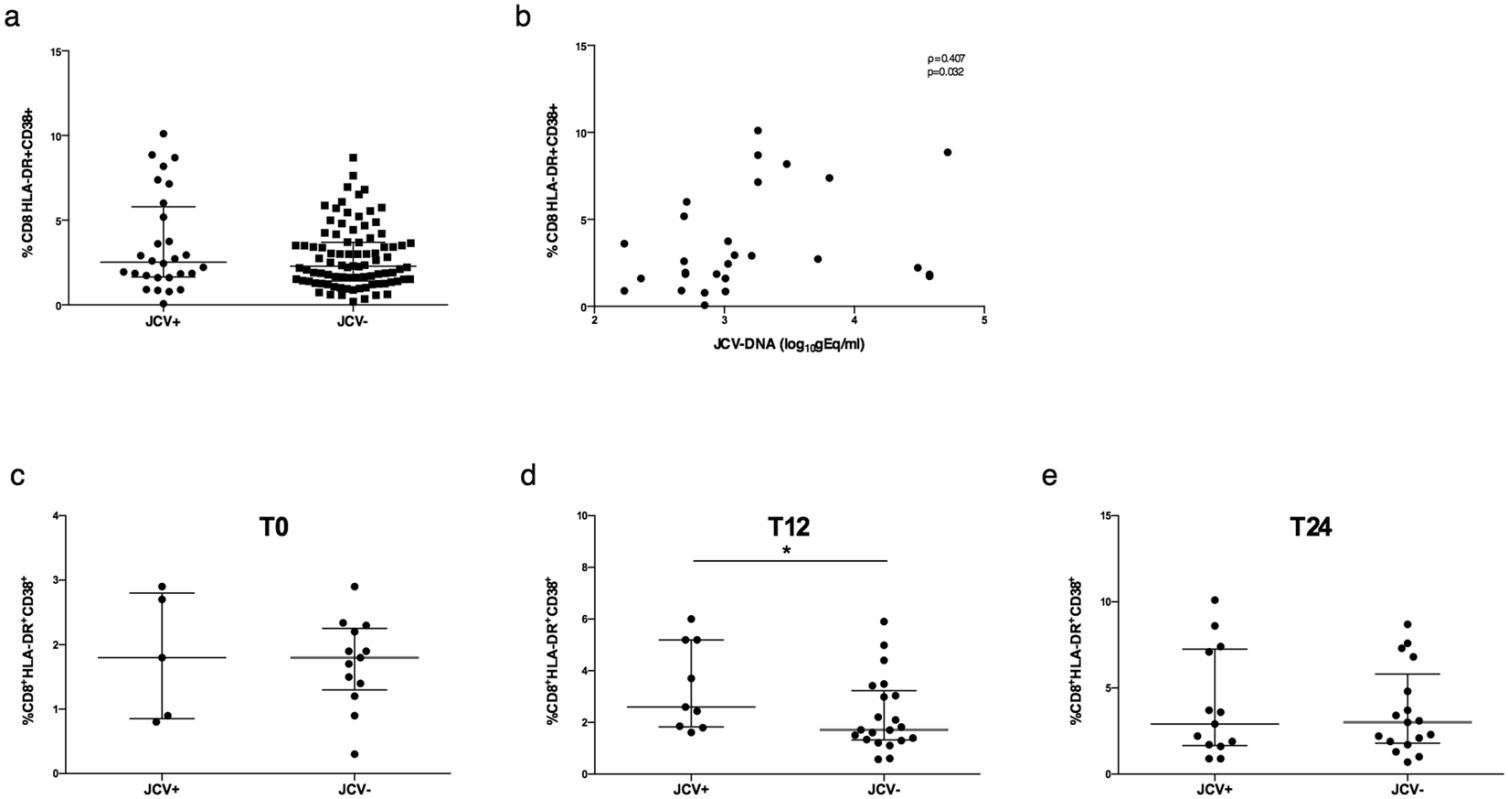
JCV viral load and CD8 immune activation levels. Evaluation of CD8 immune activation percentages in JCV+ (n = 28) and JCV− (n = 88) samples. No statistical differences were found (Mann-Whitney test). Data are shown as median (lines) and interquartile range (whiskers) (**a**). CD8^+^ T-lymphocyte immune activation percentages were correlated to JCV viral load. Correlation was performed using Spearman test (Spearman coefficient [ρ] and statistical significance [p] are reported in the graphics) (**b**). Samples were stratified into three groups: T0 (before the first natalizumab infusion) (n = 18); T12 (within the first year of treatment) (n = 30); T24 (during the second year of treatment) (n = 30). No differences were found in CD8 immune activation levels comparing JCV+ and JCV− samples in T0 (**c**) and T24 (**e**) groups; CD8 immune activation levels were increased in JCV+ compared to JCV− samples in T12 group (**d**). Data are shown as median (lines) and interquartile range (whiskers). JCV+: JCV-DNA positive samples, JCV−: JCV-DNA negative samples. *p < 0.05 (Wilcoxon test).

The explorative analysis performed considering all the 116 samples from 34 RRMS patients, showed that the median CD8 immune activation values were higher in JCV+ than JCV− samples, although the differences were not statistically significant (2.52 [1.64–5.80] and 2.28 [1.41–3.70], respectively), (Mann-Whitney, p > 0.05) (Fig. 4a). A positive correlation between CD8 immune activation percentages and JCV viral load was found (Spearman, ρ = 0.407 and p = 0.032) (Fig. 4b).

After stratifying samples according to natalizumab infusion number in T0, T12 and T24 groups, as previously described, no differences were found in CD8 immune activation levels between JCV+ and JCV− samples for T0 group (median values [IQR]: 1.80 [0.85–2.80] vs 1.80 [1.30–2.25]), (Mann-Whitney, p = 0.94) and T24 group (median values [IQR]: 2.90 [1.65–7.25] vs 3.00 [1.80–5.80]), (Mann-Whitney, p = 0.959), (Fig. 4c,e). By contrast, CD8 immune activation levels were increased in JCV+ compared to JCV− patients for T12 group (median values [IQR]: 2.60 [1.83–5.20] vs 1.71 [1.32–3.23]), (Mann-Whitney, p = 0.04) (Fig. 4d).

**Figure 5:**
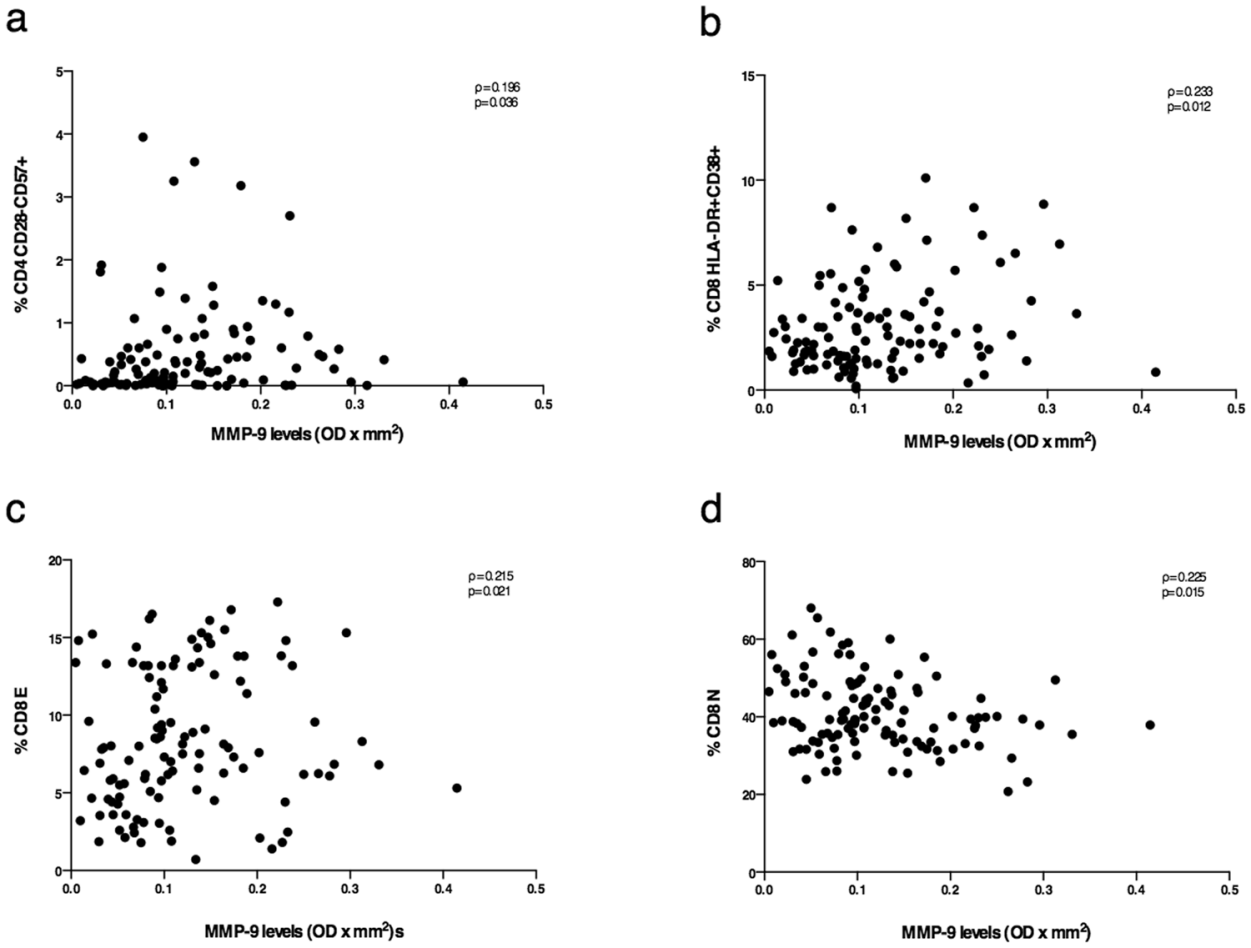
Correlation between MMP-9 plasma levels and T-lymphocyte phenotype. CD4^+^ T-lymphocyte immune senescence percentages (**a**), CD8^+^ T-lymphocyte immune activation percentages (**b**) and CD8 E percentages were positively correlated to MMP-9 plasma levels (**c**). CD8 N percentages were negatively correlated to MMP-9 plasma levels (**d**). All correlations were performed using Spearman test (Spearman coefficient [ρ] and statistical significance [p] are reported in the graphics).

Interestingly, considering MMP-9 plasma levels and immunological parameters, in 116 samples from 34 RRMS patients, CD4 T-lymphocyte immune senescence and CD8 immune activation levels were positively correlated to MMP-9 plasma levels (Spearman, ρ = 0.212 and p = 0.023; Spearman, ρ = 0.184 and p = 0.048, respectively) (Fig. 5a,b). Moreover, CD8 E percentages were positively correlated to MMP-9 plasma levels (Spearman, ρ = 0.195 and p = 0.037) (Fig. 5c), whereas CD8 N percentages were inversely correlated to MMP-9 plasma levels (Spearman, ρ = −0.231 and p = 0.013) (Fig. 5d).

## Discussion

The role of MMPs in MS pathogenesis and progression has been widely investigated. Specifically, it has been shown that the gelatinase subfamily, including MMP-2 and MMP-9, can facilitate the influx of inflammatory cells into the CNS, and contribute to the breakdown of the BBB^26^. Furthermore, gelatinases are able to cleave human myelin basic protein *in vitro*, thus playing a role in the immune pathogenesis of MS, leading to demyelination^27,28^. There is a large agreement on the pro-inflammatory role of MMP-9 in MS, considering that serum levels were found increased in patients with the RR form of MS compared to the progressive form of the disease^14^. Furthermore, MMP-9 serum levels were found increased in patients with MRI evidence of active lesions^13^ and was predictive for the appearance of gadolinium positive MRI lesions in MS subjects with secondary progressive forms^29^. It has been demonstrated that MMP-9 protein concentrations or enzymatic activity were increased in the CSF of patients with MS, especially during the evidence of either clinical or MRI active disease^30^. Moreover, some authors demonstrated that higher MMP-9 plasma levels were associated to the severity of the disease^31^.

As reported by other authors^10,11^, the current study showed higher MMP-9 plasma levels in untreated RRMS patients (T0) compared to HD, strengthening the pathogenic role of MMP-9 in MS.

In this paper, we considered MMP-9 and MMP-2 plasma levels in RRMS patients treated with natalizumab, at different number of infusions, showing that MMP-2 plasma levels remained unchanged in all RRMS patients, independently from the exposition to natalizumab treatment. Conversely, despite an initial stability in MMP-9 plasma levels up to 12 natalizumab infusions, a linear increase was observed from 12 to 24 infusions. The longitudinal analysis further confirmed the increase in MMP-9 plasma levels at 24 natalizumab infusions in comparison to samples collected from the same patients within the first 12 month of treatment.

In our cohort, the increased MMP-9 plasma levels were not associated to clinical or MRI relapses.

Recently, other authors have investigated serum levels of MMP-2 and MMP-9 active forms together with serum concentrations of tissue inhibitors of metalloproteinases (TIMP)-1 and TIMP-2 in a cohort of natalizumab treated RRMS patients. Although they did not find any increase in MMP-9 and MMP-2 active forms during natalizumab treatment and demonstrated that MMP-9/TIMP-1 and MMP-2/TIMP-2 ratios remained unchanged, an imbalance in MMP-9/MMP-2 ratio was found for RRMS patients with 15, 18 and 21 natalizumab infusions compared to RRMS patients with shorter exposition to the drug^32^. Interestingly, these data seem to be aligned to the increased MMP-9 levels observed in our cohort after the first year of natalizumab treatment.

In the present work, in order to study the influence of JCV reactivation on MMP-9 plasma levels, we stratified our samples in JCV+ and JCV−, according to JCV-DNA detection. A preliminary analysis, in which we included all the collected samples, evidenced a significant increase in MMP-9 plasma levels in JCV+ compared to JCV− samples, suggesting that JCV circulation in peripheral blood could be implicated in the increase of MMP-9 levels. This hypothesis is reinforced by the positive correlation found in our experiments between MMP-9 plasma levels and JCV viral load and by several studies that showed as MMPs are up-regulated by different infectious agents, including viruses^33^. Notably, a more detailed analysis after stratification of plasma samples into three groups (T0, T12 and T24, according to natalizumab infusion number), in which each patient contributed only with one sample, further confirmed the increase of MMP-9 plasma levels in JCV+ samples in T24 group. Accordingly, we can speculate that the elevated MMP-9 plasma levels observed in RRMS patients beyond 12 months of natalizumab treatment and in patients with detectable JCV-DNA in plasma, contribute to the increased risk of developing PML. It has been demonstrated that natalizumab can mobilize CD34^+^ precursors latently infected by JCV from the bone marrow to peripheral blood^34^. Therefore, the increased MMP-9 plasma levels can disrupt the integrity of the BBB and facilitate JCV entry into the CNS. Moreover, the impairment of the immune surveillance mechanisms in the CNS, secondary to natalizumab treatment^25^, represents another risk factor implicated in the pathogenesis of PML. In this respect, Fissolo *et al*. have recently reported decreased MMP-9 mRNA levels in PBMC and protein plasma concentrations at baseline in RRMS patients who developed PML, compared to patients who did not experience PML over 5 years of natalizumab treatment. In their study the differences of MMP-9 levels were inconstantly detected after 12 and 24 months of natalizumab treatment in pre-PML and not-PML patients^23^. In that paper, they speculate that reduced levels of molecules involved in BBB disruption, such as MMP-9, could interfere with CD8^+^ T-lymphocyte immune surveillance mechanisms in the CNS, predisposing MS patients to JCV reactivation and PML development^23^. Our results cannot be directly compared with those obtained by Fissolo *et al*., considering that in our cohort we did not observe any case of natalizumab induced PML. Moreover, Fissolo *et al*. did not evaluate plasma JCV-DNA levels in RRMS patients of their cohort.

In previous papers, we evaluated T-lymphocyte immune activation, immune senescence and maturation subsets and demonstrated that CD8^+^ T-lymphocytes immune activation percentages were increased during natalizumab treatment. Furthermore, CD4 and CD8 naïve percentages were unchanged, while CD4 and CD8 effectors increased during natalizumab treatment^25,35^. Although in this study, the analyses of all 116 samples evidenced a positive correlation between JCV viral load and CD8 immuno activation percentages, surprisingly no differences were found in the CD8 immune activation percentages between JCV+ and JCV− samples. By contrast, the longitudinal analysis in samples from 26 RRMS patients, stratified according to natalizumab infusion number, showed a significant increase of CD8 immuno activation percentages in JCV+ in comparison to JCV− samples in the T12 group. The lack of increase in CD8 immune activation percentages in JCV+ samples of T0 and T24 groups might be explain considering the low number of samples analysed and the high variability of CD8 immune activation percentages in both JCV+ and JCV− stratified samples.

To the best of our knowledge, this is the first study that analyzes the relationship between MMP-9 plasma levels and T-lymphocyte subsets in a cohort of RRMS patients under natalizumab treatment. Here we studied the correlation between MMP-9 plasma levels and T-lymphocyte phenotype in RRMS patients under natalizumab treatment and found that MMP-9 plasma levels resulted positively correlated with CD4 immune senescence, CD8 immune activation and CD8 E percentages. By contrast, a negative correlation between MMP-9 plasma levels and CD8 N percentages was found. The correlation between MMP-9 plasma levels and CD4 immune senescence underlines that these cells are not only involved in the MS pathogenesis, but may contribute to pro-inflammatory mechanisms due to their cytotoxic function and resistance to apoptosis, as previously shown by other authors^4^.

All together, these data show that T-lymphocyte immune activation is directly correlated to MMP-9 plasma levels. We could not establish whether the increased MMP-9 level is a cause or a consequence of T-lymphocyte immune activation, considering that MMP-9 is produced by and modulate the activation state of T-lymphocytes. Interestingly, in a mouse model MMP-9 seemed to be essential for the induction of T-lymphocyte proliferation and cytokine production. Specifically, the inhibition of MMP-9 and MMP-2 activity was able to abrogate anti-CD3–induced T-cell proliferation and in MMP-9^−/−^ knock-out mice, T-lymphocytes showed impairment in cytokine transcription and protein expression^36^. In agreement with these evidences, it has been shown that CD4^+^ and CD8^+^ T-lymphocytes, among other cells, have the ability to produce MMP-2 and MMP-9 upon stimulation^37^.

In conclusion, we postulate that in RRMS patients, the combined effect of natalizumab treatment and JCV reactivation could enhance MMP-9 enzymatic activity, thus contributing to CD4 and CD8 immune activation and BBB impairment. Taken together all these findings could contribute to a better understanding of PML pathogenesis in RRMS patients under natalizumab treatment, suggesting the critical role of MMP-9. More studies involving larger cohorts of patients and evaluating MMP inhibitors together with MMPs levels are advisable to explore the potential use of MMP-9 as a predictive tool for PML onset during natalizumab treatment.

## Materials and Methods

### Ethic statement

This study was approved by the Ethics Committee of Policlinico Umberto I of Rome (protocol number 130/13). All participants fulfilled the Italian Agency of Drug (AIFA) criteria for natalizumab (Tysabri®) treatment and provided a written informed consent. All procedures were performed in accordance with the guidelines and regulations of the institutional research committees and with the Declaration of Helsinki.

### Study population

A total of 116 plasma samples were collected from 34 patients affected by RRMS, enrolled from March 2013 to March 2016 at the Department of Human Neuroscience, Multiple Sclerosis Center of the University of Rome “Sapienza”.

All patients were under a natalizumab-based treatment. A “wash out” period of at least 1 month for immunomodulatory drugs and 6 months for immunosuppressive drugs was mandatory before initial natalizumab administration. According to the therapeutic protocol, an intravenous dose of 300 mg of natalizumab was administered every 4 weeks. As shown in Table 2 all samples were stratified according to natalizumab infusion number (N0: no infusion, N3: three infusions, N6: six infusions, N12: twelve infusions, N15: fifteen infusions, N18: eighteen infusions and N24: twenty-four infusions).

**Table 2:** Demographic and clinical features of RRMS patients and healthy donors.

	HD	RRMS						
		Natalizumab infusion number						
		0	3	6	12	15	18	24
Number of samples	10	18	11	14	26	10	8	29
F/M	5/5	6/12	4/7	8/6	13/13	5/5	4/4	16/13
Median age in years [IQR]	30 [27.0–34.5]	38 [32–43]	43 [40–42]	39 [38–43]	38 [32–43]	39 [29–44]	40 [36–43]	38 [33–43]
Median years of disease [IQR]	—	8 [6–10]	10 [9–12]	10 [7–13]	8 [6–10]	10 [7–17]	9 [6–14]	8 [6–11]
Median EDSS [IQR]	—	2 [1–2]	1 [1.8–2]	2 [2–3]	2 [1–2]	1.5 [1–2]	2 [1–2]	2 [1–3]
No therapy* (/N)	—	4/18	2/11	2/14	6/26	2/10	2/8	8/29
Interferon β* (/N)	—	12/18	7/11	11/14	17/26	8/10	6/8	18/29
Mitoxantrone and Interferon β* (/N)	—	1/18	1/11	1/14	1/26	0/10	0/8	1/29
Glatimer acetate* (/N)	—	1/18	1/11	0/14	2/26	0/10	0/0	2/29

HD: healthy donors; RRMS: relapsing-remitting multiple sclerosis; F: female; M: male; IQR: interquartile range; N: number of samples; EDSS: Expanded Disability Status Scale, with values ranging from 0 (normal neurological examination) to 10 (bedridden patient)^39^ *therapy before starting natalizumab.

All patients regularly underwent a complete physical and neurological examination and neurological disability was assessed with the Expanded Disability Status Scale (EDSS) score^38^. As a control group, 10 healthy donors (HD) age and sex matched with RRMS patients were enrolled.

### Sample collection

Blood samples were collected before natalizumab administration in heparin and ethylenediamine tetra-acetic acid (EDTA) tubes and plasma was obtained from whole blood after centrifugation and stored at −80 °C. Heparin plasma was used for zymography while EDTA plasma was used for viral genome detection. Heparin whole blood was used for multi-color flow cytometry immunophenotyping.

### MMP-9 plasma activity detection

As described by Liuzzi *et al*.^10^, MMP-2 and MMP-9 plasma levels were detected by zymography as white bands of digestion on the blue background of the gel and were identified by co-localization on the zymogram with human MMP-2 or MMP-9 standards (ALEXIS Biochemicals, San Diego, CA, USA). Quantitation of MMP-2 and MMP-9 levels were performed using computerized image analysis (Image Master 1D, Pharmacia Biotech, Buckinghamshire, UK) through one-dimensional scanning densitometry (Ultroscan XL, Pharmacia Biotech). MMP levels were expressed as optical density (OD) × mm^2^, representing the scanning area under the curves, which considers both brightness and width of the substrate lysis zone. An example of zymogram gelatin gel is represented in Fig. 1a.

### Viral genome detection

Viral DNA was extracted from plasma obtained from EDTA blood with the DNeasy Blood & Tissue Kit (QIAGEN, S.p.A, Milan, Italy), according to the manufacturer’s instructions. The extraction product was amplified with a real time PCR (qPCR) system using a 7300 Real-Time PCR System (Applied Biosystems, USA) and specific primers and probes, as previously described^39^.

### T-lymphocytes analysis

As previously described^25^, immunofluorescence staining for CD4+ and CD8+ T-lymphocyte subsets, immune activation and senescence, was performed using a lyse-and-wash protocol on heparin whole blood samples. Sample acquisition was performed on a MACS Quant flow cytometer (Miltenyi Biotec, Bergisch Gladbach, Germany). Flow cytometry data were analyzed using FlowJo Software v. 10.

### Statistical analyses

Statistical analyses were performed using GraphPad Prism version 6 for Mac OS X (GraphPad Software MacKiev). The 2-tailed χ^2^ test or Fisher’s exact test were used for comparing proportions. The Mann-Whitney and Kruskal-Wallis tests were used for comparing medians, as appropriate. Quantitative data are presented as median and interquartile range (IQR), unless otherwise stated. Correlations were performed through Spearman’s coefficient analysis. Linear regression was performed using the regression test. Differences were considered significant if p ≤ 0.05.

## Electronic supplementary material

Supplementary figure S1
