# Supplementary material for: Dynamic changes of MMP-9 plasma levels correlate with JCV reactivation and immune activation in natalizumab-treated multiple sclerosis patients
Source: Sci Rep. 2019 Jan 22;9:311. doi: 10.1038/s41598-018-36535-5 (PMC6342994; doi:10.1038/s41598-018-36535-5)
Supplement: Supplementary file 1 — Supplementary figure S1 [file 41598_2018_36535_MOESM1_ESM.pdf]

Dynamic changes of MMP-9 plasma levels correlate with JCV reactivation and immune activation in natalizumab-treated multiple sclerosis patients

Marco Iannetta[1, +], Maria Antonella Zingaropoli[1, +, *], Tiziana Latronico[2], Ilaria Pati[2], Simona Pontecorvo[3], Carla Prezioso[1], Valeria Pietropaolo[1], Antonio Cortese[3], Marco Frontoni[3], Claudia D'Agostino[1], Ada Francia[3], Vincenzo Vullo[1], Claudio Maria Mastroianni[1], Grazia Maria Liuzzi[2, ++], Maria Rosa Ciardi[1, ++]

[1] Department of Public Health and Infectious Diseases, Sapienza University, Rome, Italy
[2] Department of Biosciences, Biotechnology and Biopharmaceutics, Aldo Moro University, Bari, Italy
[3] Department of Human Neuroscience, Multiple Sclerosis Center, Sapienza University, Rome, Italy

[+]These authors are considered co-first authors and contributed equally to the manuscript.

[++]These authors are considered co-last authors and contributed equally to this work.

*Corresponding author: mariaantonella.zingaropoli@uniroma1.it or m.antonellazingaropoli@gmail.com

Supplementary figure S1. Evaluation of MMP-9 plasma levels according to natalizumab infusion number in 34 RRMS patients and CD8 immune activation in 26 RRMS patients.

Scattered plot of MMP-9 plasma levels according to natalizumab infusion number showed that MMP-9 plasma levels were unchanged from 0 to 12 natalizumab infusions. Conversely, MMP-9 plasma levels resulted positively correlated to the number of natalizumab infusions from 12 to 24 months of treatment.

Correlations were performed using the non-parametric Spearman test (Spearman coefficient [ρ] and statistical significance [p] are reported in the graphics). Linear correlation was evaluated by using the regression test, R^2=0.063, p=0.032 (a).

The zymogram gel represents MMP-9 and MMP-2 plasma levels from three different healthy donors (HD) with high (lane 1) and low (lanes 7 and 10) MMP-9 levels. Lanes 2-6 and 8, 9 show MMP-9 and MMP-2 plasma levels from the same RRMS patient in samples collected at 0, 3, 6, 12, 15, 24 and 18 natalizumab infusions, respectively (b).

Longitudinal evaluation of $CD8^+HLA\text{-}DR^+CD38^+$ percentages in 26 RRMS patients. Whole blood samples were collected within 12 (T≤12) and at 24 (T24) natalizumab infusions. For T≤12 group, all samples collected at T0 (before first natalizumab infusion) were included in the analysis. When a T0 sample was not available, the first sample collected within 12 natalizumab infusions was considered. $CD8^+HLADR^+CD38^+$ median percentages [IQR]: 1.64 [1.25-2.22] for T≤12 and 2.97 [1.71-6.26] for T24, Wilcoxon p=0.004 (c).

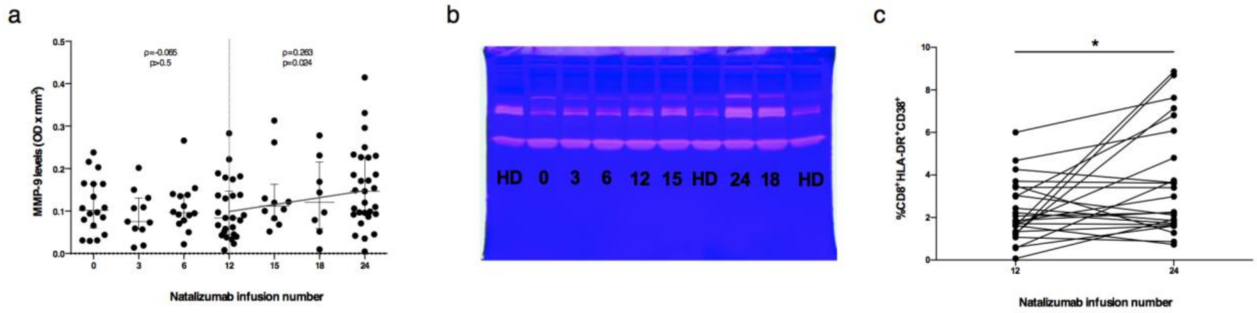
a
MMP-9 levels (OD x mm2)
0.0
0.1
0.2
0.3
0.4
0.5
ρ=-0.065
p>0.5
ρ=0.263
p=0.024
0
3
6
12
15
18
24
Natalizumab infusion number
b
HD
0
3
6
12
15
HD
24
18
HD
c
%CD8+HLA-DR+CD38+
0
2
4
6
8
10
*
12
24
Natalizumab infusion number
